# MS-MLPA analysis for *FMR1* gene: evaluation in a routine diagnostic setting

**DOI:** 10.1186/1471-2350-14-79

**Published:** 2013-08-05

**Authors:** Valentina Gatta, Elena Gennaro, Sara Franchi, Massimiliano Cecconi, Ivana Antonucci, Marco Tommasi, Giandomenico Palka, Domenico Coviello, Liborio Stuppia, Marina Grasso

**Affiliations:** 1 Laboratory of Molecular Genetics, Department of Psychological, Humanities and Territorial Sciences, School of Medicine and Health Sciences, “G. d’Annunzio” University, via dei Vestini 31, Chieti, 66013, Italy; 2 Aging Research Center, “G. d’Annunzio” Foundation, Chieti –Pescara, via Colle dell’Ara, Chieti, 66013, Italy; 3 Laboratory of Genetics, Galliera Hospital, via A. Volta 8, Genoa, 16128, Italy; 4 Department of Medical Sciences Oral and Biotechnologies, “G. d’Annunzio” University, via dei Vestini 31, Chieti, 66013, Italy

**Keywords:** *FMR1*, Fragile X Syndrome, Methylation, MS-MLPA

## Abstract

**Background:**

Fragile X Syndrome (FXS), the most common cause of familiar mental retardation, is associated in over 99% of cases to an expansion over 200 repeats of a CGG sequence in the 5’ UTR of the *FMR1* gene (Xq27.3), leading to the hypermethylation of the promoter. Molecular diagnosis of FXS have been so far based on the use of the Southern Blot (SB) analysis, a low throughput and time consuming technique. In order to update the diagnostic approach for FXS, we evaluated the usefulness of the Methylation-Specific Multiplex-Ligation-dependent Probe Amplification assay (MS-MLPA).

**Methods:**

The study was carried out by retrospectively analysing 44 male patients, 10 Chorionic Villus Sampling (CVS) samples and 10 females previously analyzed by SB. In addition, a prospective study on 98 male subjects, 20 females and 1 CVS sample was carried out for assessing the feasibility and the impact of MS-MLPA in a routine lab work.

**Result:**

Results provided by both the retrospective and the prospective parts of this study strongly demonstrate the robustness and reproducibility of the MS-MLPA assay, able to correctly detect the methylation status in all normal and full mutation male samples analyzed, including CVS male samples. On the other hand, MS-MLPA analysis on females samples produced unreliable results.

**Conclusion:**

Based on our results, we suggest the necessity of a separate workflow for male and female patients with suspected FXS in the routine diagnostic setting. MS-MLPA, in combination with CGG repeat sizing using a single-tube primed *FMR1* PCR, represents a reliable diagnostic protocol in the molecular diagnosis of FXS male patients.

## Backround

Fragile X Syndrome (FXS) (OMIM #300624) is a common cause of familial mental retardation (MR) and the second cause of mental impairment after trisomy 21 in males, representing about 1% of all MR and 10% of X-Linked MR [1]. Recent epidemiological studies indicate that FXS is responsible for moderate to severe mental retardation in 1:4000–6000 males of European descent; the same condition is also responsible for mild-to-mo-derate mental retardation in 1:7000–10000 females [2,3]. In affected boys, delay in language acquisition and/or behavioural problems with frequent occurrences of autistic-like features and hyperactivity are the main presenting symptoms. The characteristic clinical signs, such as mild facial dysmorphism with long face and large ears, and macroorchidism, are established around puberty. In the majority of cases (99%), the disease is associated with an expansion over 200 repeats of the CGG sequence located in the 5’ UTR of the *FMR1* gene (Xq27.3), leading to the hypermethylation of the promoter. As a consequence, the gene is transcriptionally silenced and the gene product, the fragile X mental retardation protein (FMRP), is absent [4]. Only repeat sizes over 200 are associated with full-blown MR as a consequence of the above described mechanism, while premutations (55–200 repeats) do not affect promoter methylation and can lead to fragile X-associated Tremor/Ataxia syndrome (FXTAS) and premature ovarian failure (POI) in females [4]. Accurate sizing of premutations by PCR can be hampered by the fact that, despite the availability of several specific techniques, large expansions are refractory to PCR amplifications, making alleles above 120 CGGs difficult to detect. As a consequence, so far the largest allele that has been amplified by PCR consists of 250 CGG repeats [5], and the amplification of alleles larger than 100 repeats is highly variable. The SB technique is still considered as the gold standard for the molecular diagnosis of FXS, being able to clearly distinguish between full mutated and premutated alleles and, by digesting DNA with methylation sensitive enzymes, providing information also regarding the *FMR1* promoter methylation status [6]. However, SB is time-consuming, it requires large amounts of DNA and possibly the use of radioactive material. Thus the first step in the molecular diagnosis of FXS in male patients is represented by a PCR-based analysis, followed by SB on in cases in which failure of PCR amplification suggests the presence of a CGG expansion. However, this approach is sometimes uninformative in females, where the presence of a single PCR amplification product may indicate either a condition of homozygosity for two FMR1 alleles with the same number of CGG repeats or the presence of a normal and an expanded allele. Recently, a novel, single-tube CGG repeat primed *FMR1* PCR (RP-PCR) technology has become available based on the use of two gene-specific primers flanking the triplet repeat region and a third primer complementary to the (CGG)n repeat. This approach provides robust detection of expanded alleles and resolves allele zygosity, thus minimizing the number of samples requiring SB analysis and producing more comprehensive *FMR1* genotyping data than other methods [7,8]. Since the distinction between a premutation and a full mutation is related to the methylation status rather than to the exact size of the repeat, in the present study we evaluated the usefulness of the Methylation-Specific Multiplex-Ligation-dependent Probe Amplification (MS-MLPA) assay to assess the methylation status of the promoter of the *FMR1* gene for the molecular diagnosis of FXS. In fact MLPA represent a widely used technique in the study of gene copy number but also for the assessment of the methylation status of specific genes [9-14]. This approach was used in a retrospective study on 44 males, 10 Chorionic Villus Sampling (CVS) samples from male foetuses and 10 females in order to verify if MS-MLPA could replace SB in the evaluation of the methylation status of the promoter of *FMR1* gene. Moreover, a prospective study on 98 male samples, 20 females and 1 CVS sample was carried out, aimed to the assessment of the feasibility and the impact of such technique in a routine lab work.

## Methods

### Patients

The study included 64 retrospective and 119 prospective samples selected according to clinical criteria and entered in routine diagnosis at the Laboratory of Human Genetics of Genoa from April to June 2011 (Table 1). Among retrospective cases, 28 (23 males, 5 females) were FXS patients carriers of a *FMR1* full mutation, 2 (1 male and 1 female) were premutated subjects and 21 (17 males and 4 females) were normal controls. DNA was obtained from peripheral blood cells using standard procedures. In addition, 10 CVS from male foetuses with gestational age ranging from 12 to 17 weeks entered this study, of which six normal and 4 full mutated male foetuses. In order to verify the analytical sensitivity of MS-MLPA technique in the detection of methylation mosaicism cases, we also tested: i) one FXS patient with a size mosaicism of premutation/full mutation (pre/full); ii) one FXS male showing a partially (near to 5%) methylated full mutation; iii) 6 additional DNA samples consisting of mixtures of dilution series (100, 75, 50, 25, 10, 5, and 2.5%) of DNA from a full-mutated patient mixed with DNA from a normal male. The fragile X genotype of each sample had been previously assessed using SB and PCR analysis, and MS-MLPA analysis was performed in blind.

**Table 1:** Samples entering in the retrospectives and prospective MLPA study

**MALES**	**Retrospectives**	**Prospectives**	**Total**
Normal	17	94	111
Premutation	1	-	1
Full Mutation	23	4	27
Size Mosaicism (Pre/Full)	1	-	1
Methylation Mosaicism	1	-	1
*FMR1*-*FMR2* deletion	1	-	1
TOTAL	44	98	142
**FEMALES**	**Retrospectives**	**Prospectives**	**Total**
Normal	4	8	12
Premutation	1	5	6
Full Mutation	5	7	12
TOTAL	10	20	30
**CVS MALE Foetuses**	**Retrospectives**	**Prospectives**	**Total**
Normal	6	1	7
Full Mutation	4	-	4
TOTAL	10		11

Finally, to test the MLPA analytical sensitivity in copy number determination in the FXS critical region in deletion cases, we analyzed a previously described patient [15], who carries a microdeletion (approx. 3,2 Mb) involving bands Xq27.3-28 and encompassing *SLITRK2*, *FMR1* and *FMR2* (*AFF2*) genes.

The 119 novel prospective samples were represented by 98 intellectually disabled males and 1 CVS from male foetus with suspected FXS and 20 females ( 7 full mutated, 5 premutated and 8 normal controls) (Table 1). In the prospective study, methylation status was investigated by MS-MLPA, followed by SB study to confirm the results.

The present study is focused on evaluating a diagnostic procedure and was approved by patients or their guardians with a written informed consent. No additional study has been performed on this material.

### MS-MLPA analysis

MS-MLPA analysis was carried out using the SALSA ME029-B1 *FMR1*/*AFF2* probemix (MRC-Holland) containing 27 probes specific for *FMR1* and *AFF2 (FMR2)* genes according to the recommendations of the manufactures [16]. Fourteen probes in the mixcontain a HhaI recognition site, 7 of which are specific for the FMR1 promoter. In addition, 13 different reference probes specific for genes at other locations are present in the kit. Each MS-MLPA reaction generates two products: one undigested product for copy number detection and one digested product for methylation detection. After MS-MLPA reaction, the samples were loaded onto an ABI 3130xl (Applied Biosystems) using POP7.

### MS-MLPA data analysis

Signals produced by MS-MLPA reactions were captured by Gene Mapper 3.2 and the specific peak area values were reported in an Excel spreadsheet. The presence of aberrant methylation was identified in these samples by the appearance after *HhaI* digestion of a signal peak that was absent in unmethylated normal controls. The diagnosis of *FMR1* deletions was based on the absence of the *FMR1* specific peaks in presence of controls peaks in undigested samples. Data Analysis was performed using the Coffalyser software v. 9.4, able to calculate the normalized ratios of *HhaI* digested to undigested peaks for each of the *FMR1* methylation-specific probes present in each sample, according to Nygren [16]_._

### SB analysis

In all patients, both in retrospective than in prospective analysis, the FRAXA locus was analyzed with conventional Southern analysis of genomic DNA (7 μg) digested with the restriction enzymes *EcoRI* and *EagI*. The blotted membranes were probed with a [32P]dCTP-labeled (Re-divue; GE Healthcare Europe) StB12.3 probe that hybridizes to the region from nucleotide 14461 to 15537 in *FMR1* (GenBank reference sequence L29074) [17].

## Results

**Figure 1:**
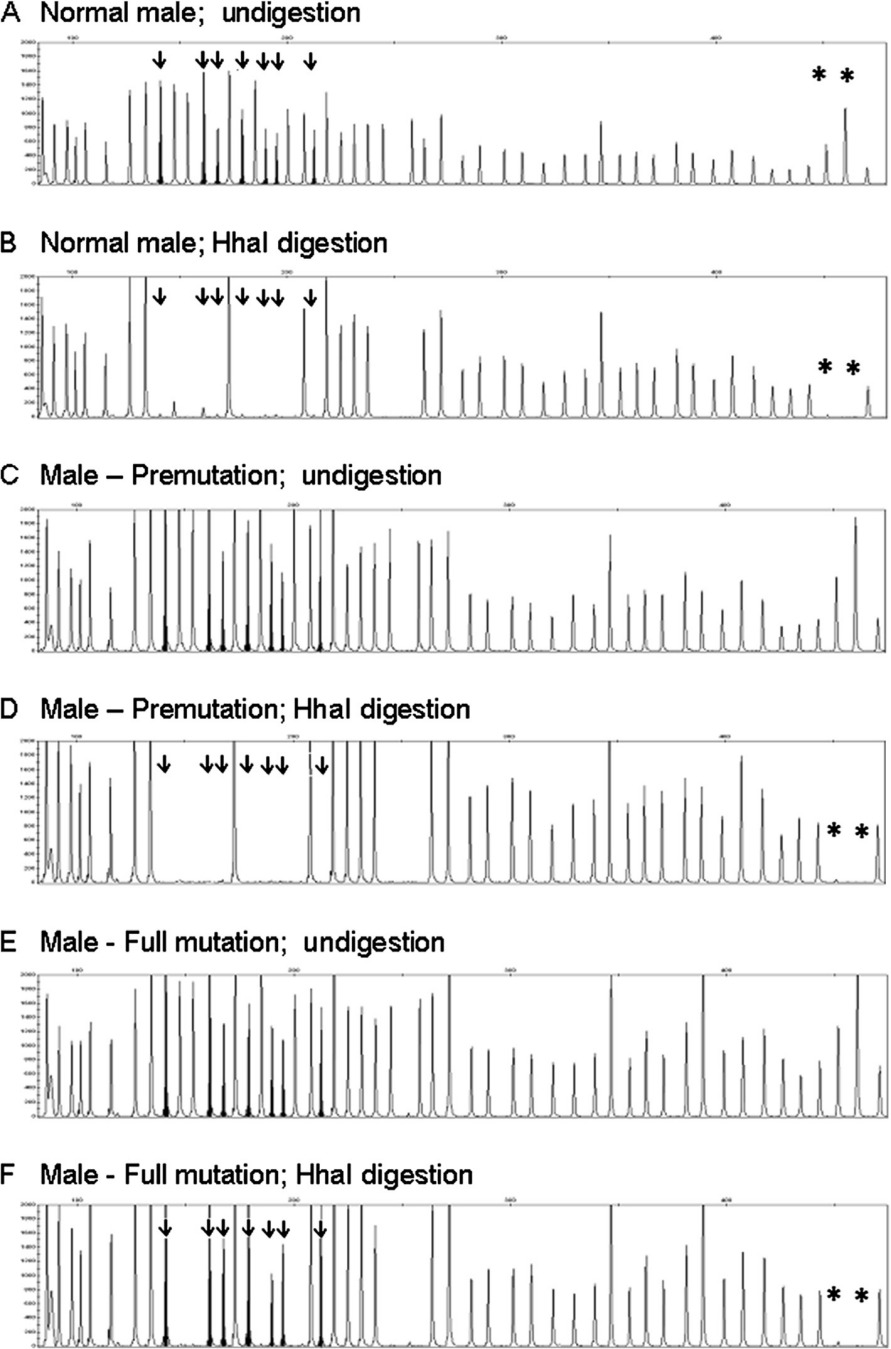
**Electropherograms showing the results of MS-MLPA analysis of the *FMR1 *gene: A,B = normal male; C,D = premutated male; E,F = full mutated patient.** Straight arrows = methylation specific *FMR1* probes; Asterisks = digestion control probes.

**Figure 2:**
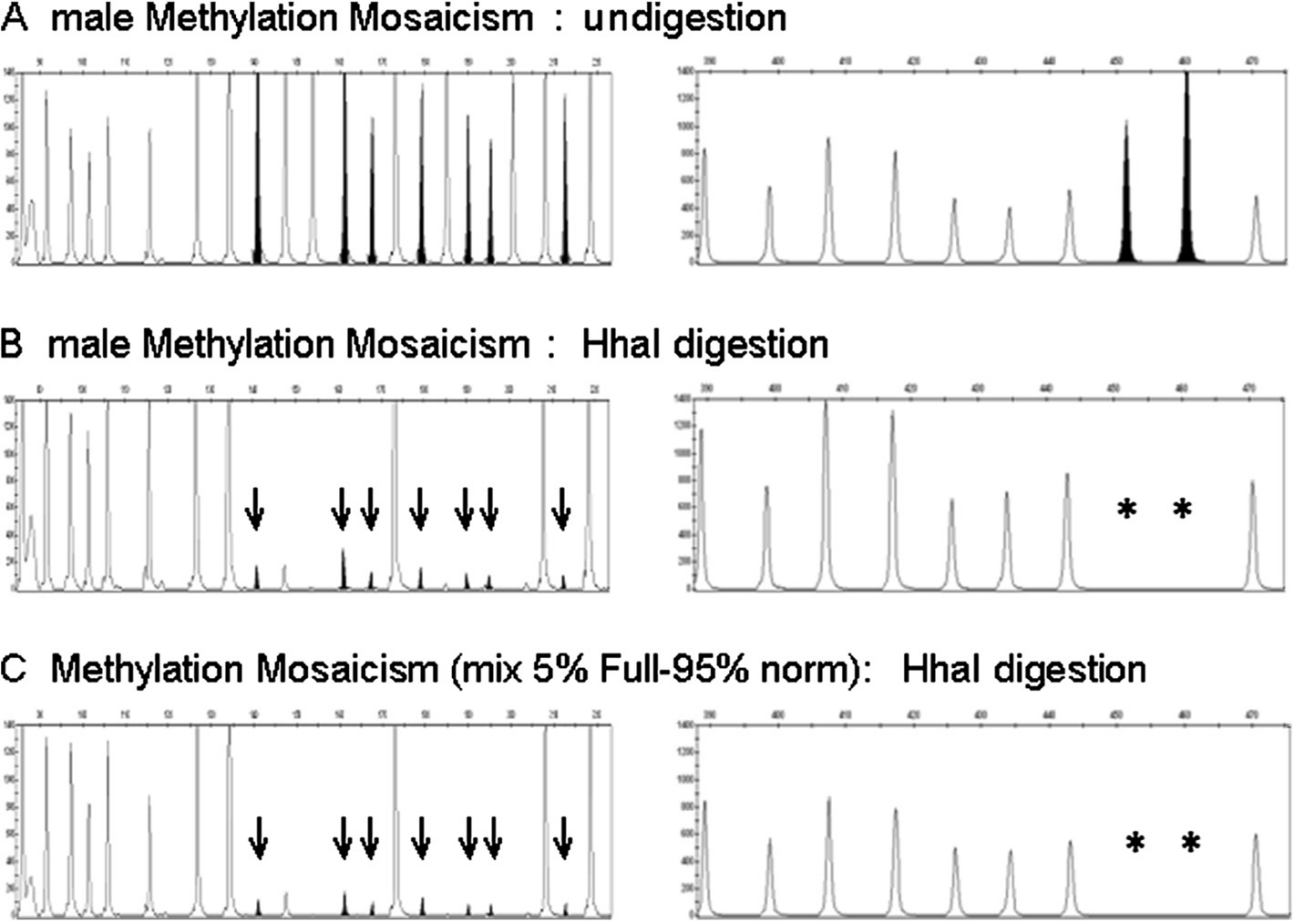
**Electropherograms showing the results of MS-MLPA analysis of the *FMR1 *gene: A = male methylation mosaic, undigested; B = male methylation mosaic after HhaI digestion; C = mixture of DNA from a full-mutated patient (5%) mixed with DNA from a normal male (95%).** Left: methylation specific *FMR1* probes (straight arrow). Right: digestion control probes (asterisks).

In all control samples and in the premutated male sample, MLPA analysis evidenced a peak ratio close to zero for all the methylation sensitive probes, thus demonstrating the absence of methylation in the *FMR1* promoter (Figure 1A-D; Table 2). In FXS male patients and in CVS samples from full mutation fetuses, all FMR1 methylation sensitive probes showed a peak ratio value > 0.64 (Figure 1E,F; Table 2), indicating the presence of an aberrant methylation of the *FMR1* promoter, concordant with the diagnosis of FXS previously assessed by SB analysis. In the patient showing a size mosaicism (pre/full), all the methylation sensitive probes of *FMR1* showed a peak ratio ranging between 0.48 and 0.85 (Table 2); in this case densitometric analysis of SB had shown that the signal of the methylated full mutation was > 84% and the unmethylated premutation was about 16% (data not shown). In the patient carrier of a methylation mosaicism, MS-MLPA analysis evidenced a peak ratio of *FMR1* methylation sensitive probes ranging between 0.04 and 0.08 (Figure 2A,B; Table 2). In this particular case the SB analysis had shown that the unmethylated full mutation smear was predominant over the methylated one,that (as evidenced by densitometric analysis) was lower than 5% (data not shown).

**Table 2:** Average ratios observed in different genotypes

***Sample type***	***Retrospective cases***	***Prospective cases***	FMR1 D*:U** range	***FMR1 D:U Average (Averege-SD)***	***Theoretical FMR1 D:U***	***CI 95% (l. inf. – l. sup.)***
**Males unaffected controls**	17	94	0-0.05	0.005 (0.017)	0	0.003-0.005
**Males premutation cases**	1	-	0-0.02	NA	0	
**Males full mutation cases**	23	4	0.64-1.37	0.89 (0.26)	1	0.867-0.951
**Males premut/Full mosaic**	1	-	0.48-0.85	0.66 (NA)	0-1	NA
**Males methylat. mosaic (5% Full)**	1	-	0.04-0.08	0.06 (NA)	0-1	NA
**Male foetuses (CVS) normal**	6	1	0	NA	0	NA
**Male foetuses (CVS) Full mut.**	4	-	0.48-1.26	0.73 (0.36)	1	0.609-0.883
**Female normal e premutat.**	5	13	0.05-0.97	0.46 (0.10)	0.5	0.412-0.508
**Female Full mutation cases**	5	7	0.1-1.73	0.48 (0.20)	0.75	0.350-0.614

*: digested with *HhaI*; ** undigested.

On the other hand, MS-MLPA analysis on females samples produced unreliable results, due to the presence of an inactive, methylated X-chromosome generating peak ratios overlapping among normal and full mutated (Table 2).

To test the MLPA detection limit in the evaluation of the methylated DNA levels, we performed a MS-MLPA testing on a dilution series of samples ranging from 100% to 2.5% of DNA from a full mutation patient. This experiment showed a proportional decrease of the peak ratio value down to 5% of methylated DNA, while the results obtained with the 2.5% dilution were not repro-ducible (Figure 2C; Table 3). These results show that methylated full mutation must represent at least 5% of the total DNA in a sample to be detected by MS-MLPA, indicating that this tool has an analytical sensitivity lower than 100%, in agreement with previous results reported by Abdool for the detection of mosaicisms of copy number variation [18]_._

**Table 3:** MS-MLPA analytical sensitivity testing: analysis of the samples dilution series ranging from 100% to 2.5% of DNA from a full-mutated patient

**Mixtures of dilution series**	*FMR1 *D*:U** range	**CI 95% (l. inf. – l. sup.)**
**75%**	0.47 – 0.88	0.432- 0.745
**50%**	0.32 – 0.65	0.316- 0.521
**25%**	0.17 – 0.3	0.178- 0.270
**10%**	0.06 – 0.14	0.323- 0.780
**5%**	0.04 – 0.09	0.111- 0.506
**2.5%**	0.00-0.08	0.001-0.062

*: digested with *HhaI*; ** undigested.

**Figure 3:**
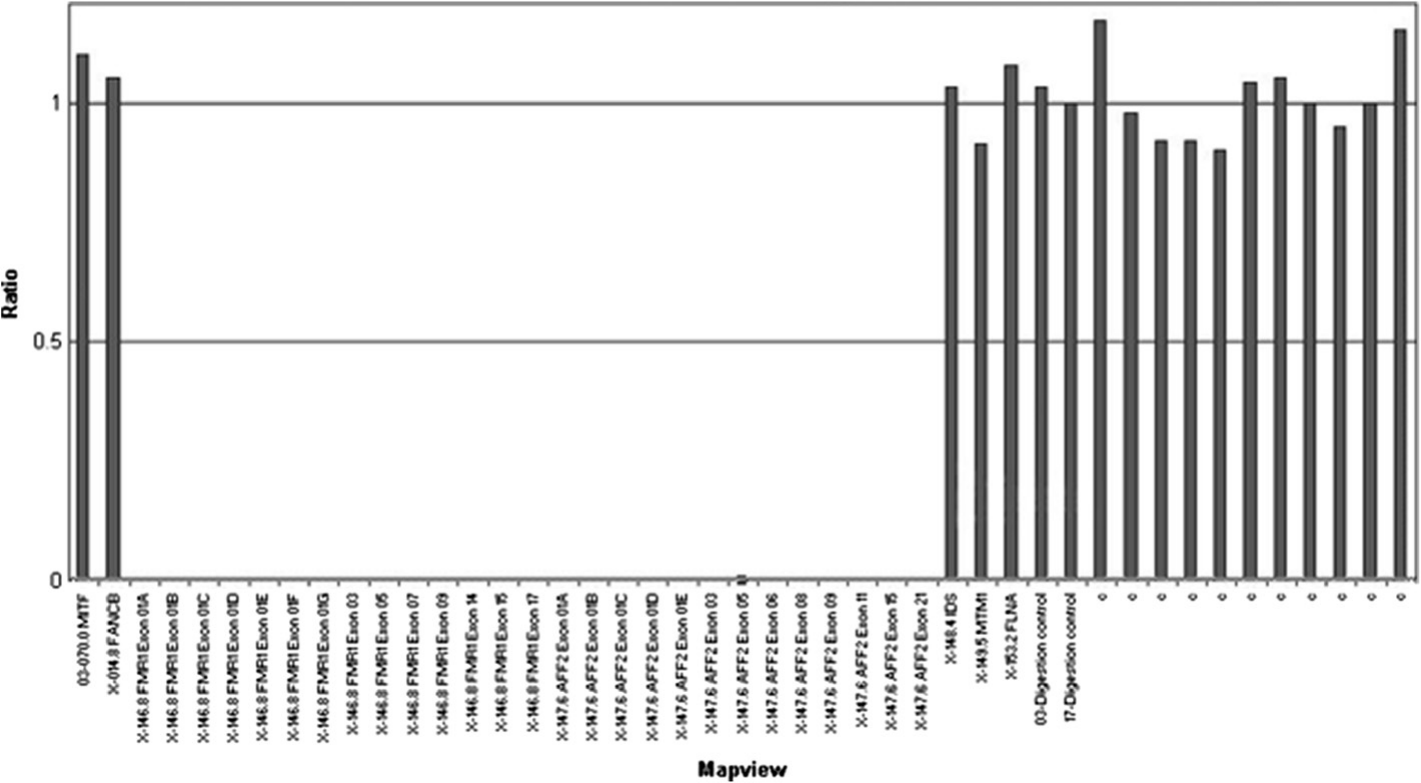
**MLPA Coffalyzer analysis: histogram shows a peak ratio = 0 in the deleted probes in the patient carrier of a microdeletion of 3.2 Mb encompassing SLITRK2, *FMR1 *and *FMR2 *genes.**

Finally, in order to test the MS-MLPA sensitivity on deleted cases, we analysed a FXS patient carrier of a deletion detected by SB and validated by CGH [18]. MLPA analysis confirmed the deletion showing a peak ratio = 0 of all specific probes for *FMR1* and *FMR2* (*AFF2*) genes (Figure 3).

## Discussion

The molecular diagnosis of FXS is a genetic routine test increasingly required since the clinical diagnosis based on dysmorphic features is quite subtle, especially in early life. The SB analysis, so far considered the gold standard test for both sizing of CGG repeat and assessing the methylation status of the *FMR1* gene promoter, is limited by the necessity of large amounts of patient DNA, the use of radioactive material and the low throughput. In order to update our diagnostic approach to FXS, we set up an internal procedure to validate the commercial kit MS-MLPA SALSA ME029-B1 FMR1/AFF2, designed to investigate both the methylation status of the FMR1 gene promoter and the gene copy number of the FRAXA locus. Results provided by both the retrospective and the prospective parts of this study demonstrate the robustness and reproducibility of the MS-MLPA assay, that correctly measured the methylation status in all normal and full mutation male samples analyzed, including CVS male samples. Thus, MS-MPLA can be considered as a useful approach in the routinely molecular testing of the *FMR1* gene, also considering that this assay represents a faster, easier, low cost and high throughput technique as compared to SB. However, the limits of MS-MLPA approach in some specific cases must be discussed. The most crucial issue related to the use of MS-MLPA assay remains the possible presence of methylation mosaicisms. Our data indicate that MS-MLPA is able to detect the presence of a methylation mosaicism only when the methylated DNA represents at least 5% of the total DNA in a sample, according to a previous report [16]. Thus, other techniques, such as SB or mPCR are required in order to assess the presence of very low level mosaicisms. On the other hand, the size mosaicism appears to be correctly detectable by MS-MLPA assay, which in our case produced an expected intermediate peak ratio ranging from 0.48-0.85, according to the presence of 85% full mutation.

Another limit of MS-MLPA is related to its application in the study of *FMR1* in females, where the random X inactivation leads to results which are often borderline, as previously reported [16].

## Conclusion

Based on these results, we suggest the necessity of a separate workflow for male and female patients with suspected FXS in the routine diagnostic setting. In the male patients we propose at first a PCR to evaluate the presence of the specific fragment amplification and its size calling. In the absence of amplification, when it is necessary to analyse the gene methylation status, MS-MLPA represents a reliable diagnostic protocol able to replace SB in the majority of cases, except those showing low levels methylation mosaicism. Since the MS-MLPA kit ME029-B1 *FMR1*/*AFF2* contains probes specific to some other exons of the *FMR1* gene as well as probes specific to *FMR2* gene, this probe mix is also useful to identify the rare cases of deletions involving *FMR1* gene, as well as methylation status and deletions of *FMR2* (*AFF2*) gene (*FRAXE*), providing a gain in the diagnostic sensibility.

Also in female patients workflow the first step is represented by PCR analysis for evidencing and eventually sizing the presence of the two amplification fragments. In case of only one amplification fragment it will be necessary to discriminate between an homozygous wild type allele or the presence of a normal allele and a pre- or full- mutated one. Since MS-MLPA, as previously discussed, is not reliable for these purposes in females, the second step can be represented by classical SB or by the recently proposed mPCR approach, combining allele-specific methylation PCR and capillary electrophoresis, producing results concordant with corresponding SB analyses [19].

In conclusion, the diagnostic scheme of FXS could be modified as described, using Southern blotting only in the few cases in which is really necessary and performing PCR followed by MS-MLPA when a large number of patients needs to be analyzed in a routine setting, in order to reduce both costs and time of the molecular analysis. In this view, laboratories not performing Southern blotting should be well aware of the limitations of the approach based on PCR/MLPA only, and should be able to recognize cases to be referred to other labs capable of doing Southern blotting.

## Competing interests

The authors declare that they have no competing interests.

## Authors’ contributions

VG: contributed to conception and design, MLPA data analysis and drafted the manuscript. EG: responsible of clinical data and DNA extraction, SF performed the FMR1 gene study through MLPA; MC responsible of clinical data and DNA extraction; IA performed the FMR1 gene study through MLPA; MT performed statistical analysis; GP contributed in the design of the study and to the MLPA data analysis; DC conceived of the study, and participated in its design; LS have given final approval of the version to be published; MG conceived of the study, and participated in its design and coordination, MLPA data analysis and helped to draft the manuscript. All authors read and approved the final manuscript.

## Pre-publication history

The pre-publication history for this paper can be accessed here:

http://www.biomedcentral.com/1471-2350/14/79/prepub
